# Hierarchically Macroporous Graphitic Nanowebs Exhibiting Ultra-fast and Stable Charge Storage Performance

**DOI:** 10.1186/s11671-018-2456-y

**Published:** 2018-02-02

**Authors:** Young Soo Yun

**Affiliations:** Department of Chemical Engineering, Kangwon National University, Samcheok, 245-711 South Korea

**Keywords:** Macroporous carbon, Carbon nanofiber, Carbonization, Electrode, Supercapacitor

## Abstract

The macro/microstructures of carbon-based electrode materials for supercapacitor applications play a key role in their electrochemical performance. In this study, hierarchically macroporous graphitic nanowebs (HM-GNWs) were prepared from bacterial cellulose by high-temperature heating at 2400 °C. The HM-GNWs were composed of well-developed graphitic nanobuilding blocks with a high aspect ratio, which was entangled as a nanoweb structure. The morphological and microstructural characteristics of the HM-GNWs resulted in remarkable charge storage performance. In particular, the HM-GNWs exhibited very fast charge storage behaviors at scan rates ranging from 5 to 100 V s^−1^, in which area capacitances ranging from ~ 8.9 to 3.8 mF cm^−2^ were achieved. In addition, ~ 97% capacitance retention was observed after long-term cycling for more than 1,000,000 cycles.

## Background

Multidimensional carbon-based nanomaterials (MCNs) have a great potential in energy storage, because of their unique material properties, such as large specific surface area, high surface to volume ratio, high electrical conductivity, and chemical/thermal/mechanical stability [1–3]. In addition, abundant and inexpensive precursor materials with well-known simple chemistries make MCNs more attractive as electrode materials for a range of energy storage devices [4, 5]. In particular, MCNs have been considered as a suitable electrode material for supercapacitors that can deliver much higher power than other energy storage devices because of their intrinsic charge storage mechanism based on physical adsorption/desorption on the surface of the electrode materials without solid state diffusion [6]. In a general point of view, the power characteristics of supercapacitors are affected strongly by the operating cell voltage, as shown in the following formula: *P*_max_ = *V*_*i*_^2^/(4*R*), where *P*, *V*_*i*_, and *R* are the power density, initial voltage, and equivalent series resistance (ESR), respectively [7]. In addition, the energy density is also closely related to the cell voltage from the relationship, *E* = 1/2 *CV*^2^, where *E*, *C*, and *V* are the energy density, capacitance, and operating voltage, respectively [8]. Therefore, a better power capability and energy density on supercapacitors can be achieved through a high cell voltage. A high working voltage of ≥ 3 V was realized using an ionic liquid-based electrolyte (ILE), while the bulky organic molecules showed unfavorable diffusion kinetics [7–9]. This suggests that a more sophisticated design of NCMs is required to maximize their electrochemical performance.

The charge storage behaviors of the MCNs are strongly dependent on both their macroscopic structure and local microstructure, particularly in ILE. Concentration polarization generally limits the rate capabilities of electrode materials due to a decline in the ion transfer ability with increasing current rates. Therefore, a hierarchically macroporous open structure composed of nanometer scale carbon building blocks can be an ideal platform to achieve rapid ion transfer; several studies reported the practicability of these architectures [10–13]. On the other hand, when ion transfer of the electrolyte is fast enough, an ohmic drop originating from inadequate electrical conductivity is a critical factor limiting the rate capabilities of the electrode materials. Graphitic carbon composed mainly of sp^2^ carbon layers generally have better electrical conductivity than amorphous carbon. Although the local carbon structures can be transformed to graphitic structures by simple heating under an inert gas atmosphere, it is difficult to maintain the internal nanostructure of the carbon-based materials during the heating process, collapsing the nanoporous architecture and/or causing the aggregation of the carbon nanobuilding blocks. Therefore, the development of advanced nanostructured graphitic materials with a large number of open macro/micropores as an electrode for high-power supercapacitors has been reported.

Bacterial cellulose (BC) is a sustainable nanofibrous polymer produced by acetic bacteria, e.g., *Acetobacter xylinum* [14, 15]. BC has a unique pore structure and properties compared to other types of cellulose in terms of its purity, high crystallinity, and high mechanical strength [15]. In our previous studies, it was found that BC pellicles can be carbonized by simple heating with maintaining its intrinsic pore structure [14, 16, 17]. And the carbonized BC pellicles were graphitized with further high-temperature heating by 2400 °C [17]. Moreover, the carbonized/graphitized BC pellicles were free-standing, which can be used as an electrode material for energy storage without binder and substrate [14, 17]. These material properties of BC could be fitted as an electrode for high-power supercapacitors.

In this study, hierarchically macroporous graphitic nanowebs (HM-GNWs) and carbon nanowebs (HM-CNWs) were prepared from BC membrane by simple heating at 2400 and 800 °C, respectively, and their electrochemical performance was characterized. The HM-GNWs possessed well-ordered graphitic microstructures including insignificant oxygen heteroatoms, showing superior electrochemical performance to HM-CNWs over a large operating voltage window of 3 V under an ILE. At a high sweep rate of 100 V s^−1^, the HM-GNWs showed a capacitance of 3.8 mF cm^−2^ and good cycling stability over 1,000,000 cycles.

## Experimental

### Preparation of HM-GNWs and HM-CNWs

BC pellicles were cultured by *Acetobacter xylinum* BRC 5 in a Hestrin and Schramm medium for 14 days. The prepared BC hydrogel was purified in an aqueous 0.25 M NaOH (97.0%, Daejung, Korea) solution and rinsed several times with pure distilled water. The neutralized BC hydrogel was then immersed in tert-butanol for 12 h at 60 °C. After freezing at − 20 °C for 5 h, the BC pellicles were lyophilized at − 45 °C and 4.5 Pa for 3 days. The resulting BC cryogels were treated thermally at 800 or 2400 °C in a graphite furnace under an Ar atmosphere at a heating rate of 5 °C min^−1^. The product HM-GNWs or HM-CNWs were stored in a vacuum oven at 30 °C.

### Electrochemical Characterization

The electrochemical properties of the samples were characterized by cyclic voltammetry (CV), chronopotentiometry, and electrochemical impedance spectroscopy (EIS, PGSTAT302N, Autolab). Ag/AgCl and Pt wire were used as the reference and counter electrodes, respectively. 1-Ethyl-3-methylimidazolium hexafluorophosphate (EMIM·PF_6_) was diluted in acetonitrile (ACN) at a weight ratio of 1:1, and the mixture solution was used as the electrolyte. The three-electrode system was tested in a beaker cell. The working electrodes are prepared by punching HM-GNWs at a diameter of 1/2 in. The loading of the active electrode was approximately 4~5 mg. The specific capacitance was determined from the galvanostatic measurements using the following equation:

1 $$ C=\frac{4{I}_{\mathrm{cons}}}{mdV/ dt}, $$

where *I*_cons_ is the (constant) current, *m* is the total mass of both carbon electrodes, and *dV/dt* was calculated from the slope of the discharge curve over the voltage window.

### Material Characterization

The morphology of the samples was characterized by field-emission scanning electron microscopy (FE-SEM, S-4300, Hitachi, Japan) and field-emission transmission electron microscopy (FE-TEM, JEM2100F, JEOL, Tokyo, Japan). The Raman spectra were recorded using a continuous-wave linearly polarized laser (514.5 nm wavelength, 2.41 eV, 16 mW power). The laser beam was focused by a × 100 objective lens, resulting in a spot ~ 1 μm in diameter. X-ray diffraction (XRD, Rigaku DMAX 2500) was performed using Cu-Kα radiation with a wavelength of 0.154 nm at 40 kV and 100 mA. X-ray photoelectron spectroscopy (XPS, PHI 5700 ESCA, USA) was used to examine the surface chemical properties of the samples. The pore structure was characterized by nitrogen adsorption and desorption isotherm tests using a surface area and porosimetry analyzer (ASAP 2020, Micromeritics, USA) at − 196 °C. The electrical conductivities of HM-CNWs and HM-GNWs were investigated using a conventional four-probe method. The punched samples were attached to gold wires using silver paint (DuPont 4929N). The *I*–*V* characteristics were measured using an electrical conductivity meter (Loresta GP, Mitsubishi Chemical, Japan). The current was applied to the samples from − 1 to 1 mA through dual sweep. The step was 0.01 mA, and each delay time is 1 s.

## Results and Discussion

**Fig. 1:**
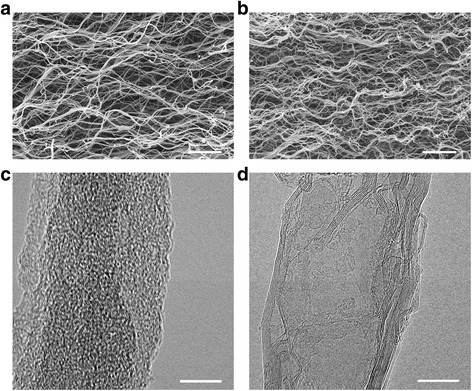
FE-SEM images of **a** HM-CNWs and **b** HM-GNWs and FE-TEM images of **c** HM-CNWs and **d** HM-GNWs. Scale bars in the FE-SEM and FE-TEM images are 2 μm and 10 nm, respectively

**Fig. 2:**
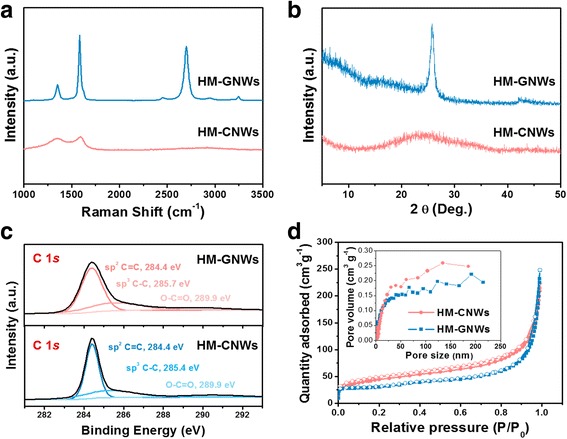
**a** Raman spectra, **b** XRD patterns, **c** XPS C 1s spectra, and **d** nitrogen adsorption and desorption isotherm curves (inset of pore size distribution data) of HM-CNWs and HM-GNWs

The morphological characteristics of the HM-CNWs and HM-GNWs were examined by FE-SEM, as shown in Fig. 1a, b, respectively. Both samples have macroporous nanoweb structures composed of entangled nanofibers with a high aspect ratio (> 100). The numerous nanofibers of both samples were approximately 20 nm in diameter and had different microstructures (Fig. 1c, d). Although HM-CNWs are composed of an amorphous carbon structure without long-range graphitic order, the HM-GNWs have highly developed graphitic structures (Fig. 1c, d). The microstructural characteristics of both samples were examined further by Raman spectroscopy and XRD, as shown in Fig. 2. The Raman spectra of the HM-GNWs showed distinct *D* and *G* bands at 1352 and 1582 cm^−1^, respectively, which correspond to the disordered *A*_1g_ breathing mode of the six-member aromatic ring close to the basal edge, and the hexagonal carbon structure related to the E_2g_ vibration mode of the sp^2^-hybridized C atoms, respectively (Fig. 2a) [7]. The sharp and split *D* and *G* bands suggest that the HM-GNWs have a well-ordered hexagonal carbon structure. In addition, the presence of a narrow 2*D* band at 2701 cm^−1^ showed that the HM-GNWs have three-dimensional ordering of the hexagonal carbon planes. In the case of HM-CNWs, the *D* and *G* bands were broad and fused to each other, indicating that they have defective carbon structures. The Raman spectrum of HM-CNWs showed no 2*D* band, which was attributed to their poor carbon ordering. In the XRD patterns, a sharp graphite (002) peak at 25.7° 2*θ* was observed for the HM-GNWs, while a highly broad peak at 24.0° 2*θ* was observed for HM-CNWs (Fig. 2b). These results coincide with the Raman spectra showing that HM-GNWs and HM-CNWs have well-ordered graphitic structures and amorphous carbon microstructures, respectively.

**Fig. 3:**
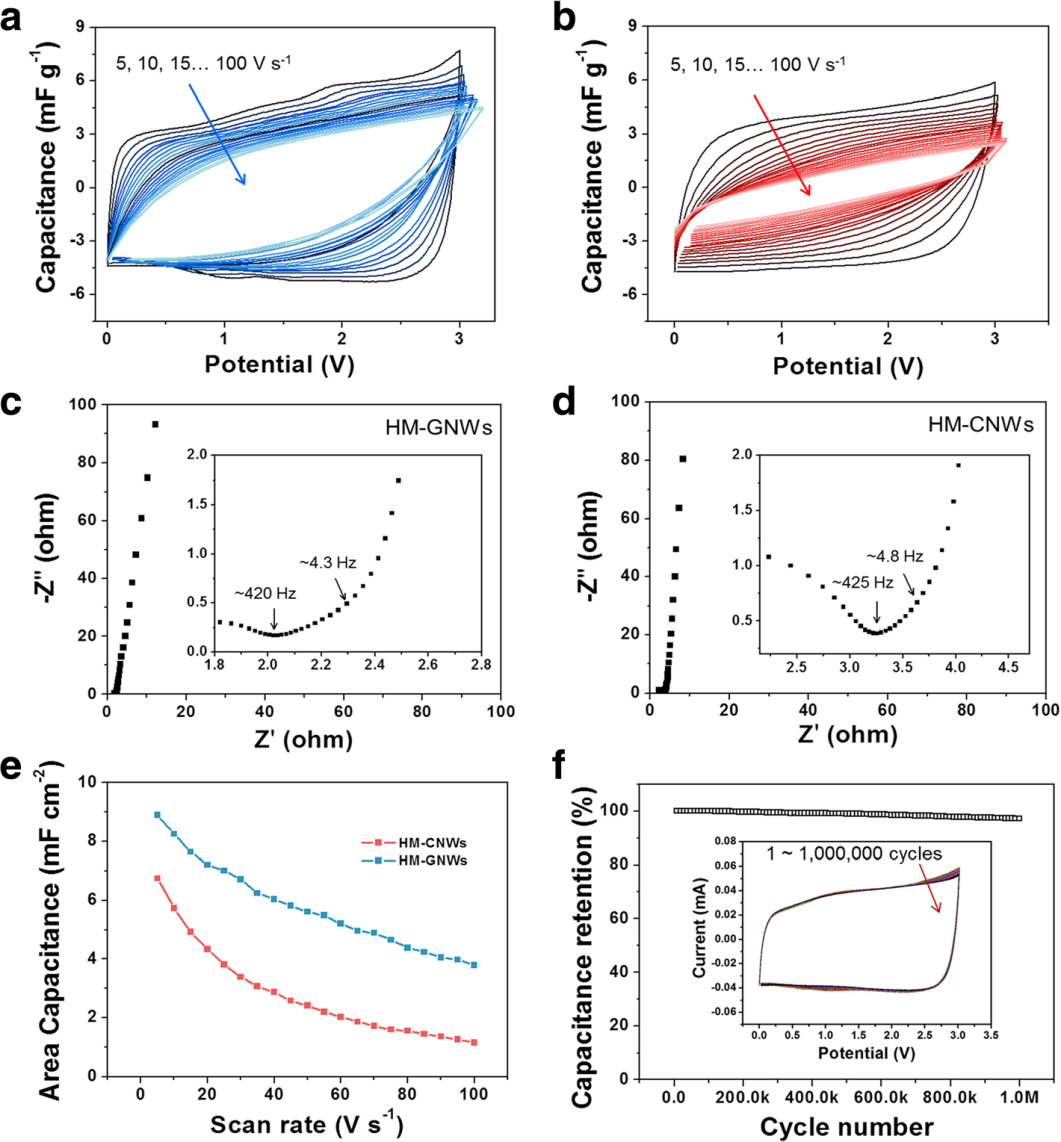
Electrochemical performance of HM-CNWs and HM-GNWs in an EMIM PF_6_/ACN mixed (1:1 *w*/*w*) solution over a voltage window of 0–3 V; CV curves at different sweep rates from 5 to 100 V s^−1^ characterized every 5 V s^−1^ of **a** HM-GNWs and **b** HM-CNWs. Nyquist plots of **c** HM-GNWs and **d** HM-CNWs (inset of the magnified images for the high frequency region). **e** Rate capabilities of both samples and **f** cycling performance of HM-GNWs (inset of the CV curves after long-term cycling)

The surface properties of the HM-CNWs and HM-GNWs were characterized by XPS, as shown in Fig. 2c. In the C 1s spectrum of the HM-CNWs, the main sp^2^ carbon bonding was observed at 284.4 eV, and two peaks, such as sp^3^ C–C and C(O)O bonding, were observed at 285.7 and 289.9 eV, respectively (Fig. 3a) [10]. Similar bonding configurations were observed in the C 1s spectra of the HM-GNWs. The C 1s spectra of HM-GNWs showed sp^2^ carbon, sp^3^ carbon, and C(O)O bonding at 284.4, 285.4, and 290.4 eV, respectively (Fig. 3c). The C/O ratios of the HM-CNWs and HM-GNWs were calculated to be 23.4 and 110.1, respectively, indicating that both samples have insignificant oxygen contents.

The pore structure of both samples was investigated using the nitrogen adsorption and desorption isotherm curves, as shown in Fig. 2d. In the isotherm curves of both samples, a small amount of monolayer adsorption of nitrogen molecules was observed in the relative pressure region of < 0.05, indicating the presence of micropores [10]. A dramatic increase in the level of nitrogen adsorption quantity was observed in the relative pressure section of > 0.9, and there was no hysteresis between the adsorption and desorption isotherm curves. These results suggest that both samples have a macroporous structure, including a small amount of micropores, corresponding to IUPAC type-I and type-II hybrid shapes. In particular, both samples have a broad range of macropore sizes which ranged from dozens of nanometers to several micrometers. The inset of Fig. 2d confirms the macropore size distribution of both samples. The specific surface areas of the HM-CNWs and HM-GNWs were 158.5 and 138.7 m^2^ g^−1^, respectively, and their pore volumes were 0.346 and 0.310 cm^3^ g^−1^, respectively.

The electrochemical performance of HM-CNWs and HM-GNWs was characterized in an EMIM PF_6_ and ACN mixed electrolyte (weight ratio 1:1) over the potential range 0–3 V (Fig. 3). CV was performed at high sweep rates from 5 to 100 V s^−1^. At a sweep rate of 5 V s^−1^, a rectangular-like CV curve was observed for the HM-GNWs, indicating ideal charge storage behaviors by the formation of a electrochemical double layer (Fig. 3a). With increasing sweep rates, the CV shapes were well maintained, even after 100 V s^−1^, which is a 0.04-s charge/discharge rate (Fig. 3a). In contrast, the CV curves of the HM-CNWs were more dented with increasing sweep rates, and the area of the CV curves was overall smaller than that of HM-GNWs, indicating the relatively poor rate capabilities of HM-CNWs (Fig. 3b). EIS of both samples characterized over the frequency range, 100 kHz to 0.1 Hz, support the CV results. The Nyquist plots of HM-GNWs and HM-CNWs exhibited a vertical line in the low-frequency region, which shows ideal capacitive charge storage behavior (Fig. 3c, d). In the high-frequency section, a transition between the RC semicircle and migration of the electrolyte was observed at a frequency of ~ 420 and ~ 425 Hz for the HM-GNWs and HM-CNWs, respectively, which correspond to a resistance of ~ 2.0 and ~ 3.3 Ω, respectively (Fig. 3c, d). The resistance of HM-GNWs was smaller than that of the HM-CNWs and much smaller than the previous results [9]. Electrolyte diffusion stopped at ~ 4.3 and ~ 4.8 Hz for the HM-GNWs and HM-CNWs, respectively; the electrochemical series resistances (ESRs) were calculated to be 2.3 and 3.7 Ω for the HM-GNWs and HM-CNWs, respectively. Therefore, both samples have low internal resistance with the HM-GNWs having the smaller value. The specific area capacitance of the HM-GNWs was ~ 8.9 mF cm^−2^ at a sweep rate of 5 V s^−1^, which decreased almost linearly with increasing sweep rates and reached 3.8 mF cm^−2^ at 100 V s^−1^ (Fig. 3e). In the case of HM-CNWs, their area capacitance at 5 V s^−1^ was 6.7 mF cm^−2^, which decreased more dramatically with increasing sweep rates. Approximately 50% of the initial area capacitance was maintained at 25 V s^−1^ for the HM-CNWs, and their area capacitance was decreased by ~ 1.1 mF g^−1^ at 100 V s^−1^. These results clearly show that the HM-GNWs have better rate capabilities than the HM-CNWs. Considering the similar porous structure and morphologies of both samples, the rate performance gap of both samples could be induced by the differences in electrical conductivity. The HM-GNWs have two orders of magnitude higher electrical conductivities (~ 130 s cm^−1^) than the HM-CNWs (~ 3.7 s cm^−1^). The cycling stability of the HM-GNWs was tested by CV at 20 V s^−1^ for more than 1,000,000 cycles, as shown in Fig. 3f. The initial capacitance was well maintained over the overall cycles, and approximately 3% of the initial capacitance was decreased after 1,000,000 cycles. These ultra-stable cycling behaviors confirm that the surface charge adsorption/desorption mechanism on the HM-GNWs is highly reversible and semi-permanent after repetitive cycling. The high rate and cycling performances of HM-GNWs were induced by their unique morphological and microstructural features based on three-dimensionally entangled graphitic nanofibers (~ 20 nm in diameter) which is much smaller and well ordered than carbon nanofibers prepared from electrospinning or template method [18–21]. Therefore, surface-induced charge storage performances can be improved in HM-GNWs, showing exceptionally high rate capabilities and cycling stabilities by sweep rates of 100 V s^−1^ and 1,000,000 cycles, respectively. The rate and cycling performances of HM-GNWs surpass them of other similar carbon-based electrode materials for supercapacitors [18–25].

## Conclusions

HM-CNWs and HM-GNWs were prepared by the pyrolysis of BC pellicles at 800 and 2400 °C, respectively. Both samples had similar macroporous nanoweb structures composed of entangled carbon nanofibers with a high aspect ratio (> 100). The HM-CNWs had an amorphous carbon structure without long range carbon ordering, whereas the HM-GNWs possessed well-ordered graphitic structures on the nanometer scale. The microstructural differences caused a considerable gap in the electrochemical performance, particularly the rate capabilities. The HM-GNWs exhibited very fast charge storage performance in an ILE, in which the area capacitance of ~ 8.9 mF g^−1^ was obtained at 5 V s^−1^, and approximately 3.8 mF cm^−2^ was achieved at an ultra-high sweep rate of 100 V s^−1^. Moreover, excellent cycling stability was observed over 1,000,000 cycles for the HM-GNWs.
